# 3-[3-Methyl-4-(3-nitro­benzyl­idene­amino)-5-sulfanyl­idene-4,5-dihydro-1*H*-1,2,4-triazol-1-yl]-1,3-diphenyl­propan-1-one

**DOI:** 10.1107/S1600536811033587

**Published:** 2011-08-27

**Authors:** Yan Gao, Yan Dong

**Affiliations:** a School of Chemical Engineering, University of Science and Technology LiaoNing, Anshan, 114051, People’s Republic of China

## Abstract

In the title mol­ecule, C_25_H_21_N_5_O_3_S, the triazole ring forms dihedral angles of 21.4 (2), 61.4 (2) and 102.4 (2)° with the nitro­phenyl and two phenyl rings, respectively. In the crystal, weak C—H⋯O hydrogen bonds and π–π inter­actions between the benzene rings from neighbouring mol­ecules [with a centroid–centroid distance of 3.571 (3) Å] consolidate the crystal packing.

## Related literature

For the crystal structures of related 1,2,4-triazole-5(4*H*)-thione derivatives, see: Al-Tamimi *et al.* (2010 ▶); Fun *et al.* (2009 ▶); Gao *et al.* (2011 ▶); Tan *et al.* (2010 ▶); Wang *et al.* (2011 ▶); Zhao *et al.* (2010 ▶).
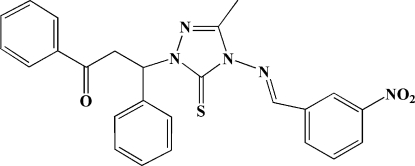


## Experimental

### 

#### Crystal data

C_25_H_21_N_5_O_3_S
*M* *_r_* = 471.53
Triclinic, 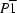

*a* = 9.0991 (10) Å
*b* = 11.8026 (15) Å
*c* = 12.0649 (16) Å
α = 70.92 (1)°
β = 73.042 (12)°
γ = 85.883 (13)°
*V* = 1170.9 (3) Å^3^
*Z* = 2
Mo *K*α radiation
μ = 0.18 mm^−1^
*T* = 113 K
0.20 × 0.18 × 0.10 mm

#### Data collection

Rigaku Saturn CCD area-detector diffractometer
Absorption correction: multi-scan (*CrystalClear*; Rigaku/MSC, 2005 ▶) *T*_min_ = 0.966, *T*_max_ = 0.983
15096 measured reflections
5553 independent reflections
3889 reflections with *I* > 2σ(*I*)
*R*_int_ = 0.031

#### Refinement

*R*[*F* ^2^ > 2σ(*F* ^2^)] = 0.034
*wR*(*F* ^2^) = 0.090
*S* = 0.98
5553 reflections
308 parameters
H-atom parameters constrained
Δρ_max_ = 0.40 e Å^−3^
Δρ_min_ = −0.17 e Å^−3^

### 

Data collection: *CrystalClear* (Rigaku/MSC, 2005 ▶); cell refinement: *CrystalClear*; data reduction: *CrystalClear*; program(s) used to solve structure: *SHELXS97* (Sheldrick, 2008 ▶); program(s) used to refine structure: *SHELXL97* (Sheldrick, 2008 ▶); molecular graphics: *SHELXTL* (Sheldrick, 2008 ▶); software used to prepare material for publication: *SHELXTL*.

## Supplementary Material

Crystal structure: contains datablock(s) global, I. DOI: 10.1107/S1600536811033587/cv5140sup1.cif

Structure factors: contains datablock(s) I. DOI: 10.1107/S1600536811033587/cv5140Isup2.hkl

Supplementary material file. DOI: 10.1107/S1600536811033587/cv5140Isup3.cml

Additional supplementary materials:  crystallographic information; 3D view; checkCIF report

## Figures and Tables

**Table 1:** Hydrogen-bond geometry (Å, °)

*D*—H⋯*A*	*D*—H	H⋯*A*	*D*⋯*A*	*D*—H⋯*A*
C3—H3⋯O1^i^	1.00	2.57	3.4922 (15)	154
C4—H4*B*⋯O3^ii^	0.99	2.59	3.5002 (16)	153
C17—H17⋯O1^i^	0.95	2.47	3.3076 (15)	147
C25—H25*B*⋯O3^iii^	0.98	2.57	3.5310 (18)	168

Symmetry codes: (i) 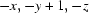; (ii) 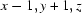; (iii) 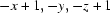.

**Fig. 1.**
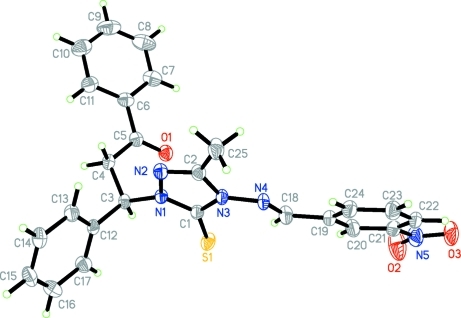
View of the molecule of (I) showing the atom-labelling scheme. Displacement ellipsoids are drawn at the 55% probability level.

## supplementary crystallographic information

### Comment

In continuation of structural study of Mannich bases derivatives synthesized by reactions of the amino heterocycles and aromatic aldehydes in our group (Wang *et al.*, 2011), we present here the crystal structure of the title compound, (I).

The bond lengths and angles in (I) (Fig. 1) are normal and comparable with those reported for the related 1,2,4-triazole- 5(4*H*)-thione derivatives (Al-Tamimi *et al.*, 2010; Fun *et al.* (2009); Tan *et al.* (2010); Wang *et al.*, 2011;). The C1 and C2 atoms in the 1,2,4-triazole ring show distorted C*_sp_*^2^ hybridization states with the bond angles of 102.22 (9)° (N1—C1—N3), 129.64 (9)° (N3—C1—S1), 110.56 (10)° (N2—C2—N3) and 126.08 (11) ° (N2—C2—C25), which are in a good agreement with the literature (Zhao *et al.*, 2010; Gao *et al.*, 2011). The 1,2,4-triazole ring makes the dihedral angles of 102.4 (2), 61.4 (2) and 21.4 (2)° with phenyl rings (C6—C11 and C12—C17) and nitrophenyl ring (C19—C24), respectively.

In the crystal structure, weak intermolecular C—H···O hydrogen bonds (Table 1) and π—π interactions between the benzene rings from the neighbouring molecules with the centroid-centroid distance of 3.571 (3) Å consolidate the crystal packing.

### Experimental

The title compound was synthesized by the reaction of 3-nitrobenzaldehyde (2.0 mmol) and 3-(4-amino-3-methyl-5-thioxo-4,5- dihydro-1*H*-1,2,4-triazol-1-yl)-1,3-diphenylpropan-1-one(2.0 mmol) by refluxing in ethnol. The reaction progress was monitored *via* TLC. The resulting precipitate was filtered off, washed with cold ethanol, dried and purified to give the target product as colourless solid in 75% yield. Crystals of (I) suitable for single-crystal X-ray analysis were grown by slow evaporation of a solution in chloroform-ethanol (1:1).

### Refinement

H atoms were positioned geometrically and refined as riding (C—H = 0.95–1.00 Å) and allowed to ride on their parent atoms, with *U*_iso_(H) = 1.2-1.5 *U*_eq_ of the parent atom.

### Crystal data

**Table:**

C_25_H_21_N_5_O_3_S	*Z* = 2
*M**_r_* = 471.53	*F*(000) = 492
Triclinic, *P*1	*D*_x_ = 1.337 Mg m^−^^3^
Hall symbol: -P 1	Mo *K*α radiation, λ = 0.71073 Å
*a* = 9.0991 (10) Å	Cell parameters from 4043 reflections
*b* = 11.8026 (15) Å	θ = 1.8–27.9°
*c* = 12.0649 (16) Å	µ = 0.18 mm^−^^1^
α = 70.92 (1)°	*T* = 113 K
β = 73.042 (12)°	Prism, colourless
γ = 85.883 (13)°	0.20 × 0.18 × 0.10 mm
*V* = 1170.9 (3) Å^3^	

### Data collection

**Table:**

Rigaku Saturn CCD area-detector diffractometer	5553 independent reflections
Radiation source: rotating anode	3889 reflections with *I* > 2σ(*I*)
multilayer	*R*_int_ = 0.031
Detector resolution: 14.63 pixels mm^-1^	θ_max_ = 27.9°, θ_min_ = 1.8°
φ and ω scans	*h* = −11→11
Absorption correction: multi-scan (*CrystalClear*; Rigaku/MSC, 2005)	*k* = −15→15
*T*_min_ = 0.966, *T*_max_ = 0.983	*l* = −15→15
15096 measured reflections	

### Refinement

**Table:**

Refinement on *F*^2^	Primary atom site location: structure-invariant direct methods
Least-squares matrix: full	Secondary atom site location: difference Fourier map
*R*[*F*^2^ > 2σ(*F*^2^)] = 0.034	Hydrogen site location: inferred from neighbouring sites
*wR*(*F*^2^) = 0.090	H-atom parameters constrained
*S* = 0.98	*w* = 1/[σ^2^(*F*_o_^2^) + (0.0481*P*)^2^] where *P* = (*F*_o_^2^ + 2*F*_c_^2^)/3
5553 reflections	(Δ/σ)_max_ < 0.001
308 parameters	Δρ_max_ = 0.40 e Å^−^^3^
0 restraints	Δρ_min_ = −0.17 e Å^−^^3^

### Special details

**Table:**

Geometry. All e.s.d.'s (except the e.s.d. in the dihedral angle between two l.s. planes) are estimated using the full covariance matrix. The cell e.s.d.'s are taken into account individually in the estimation of e.s.d.'s in distances, angles and torsion angles; correlations between e.s.d.'s in cell parameters are only used when they are defined by crystal symmetry. An approximate (isotropic) treatment of cell e.s.d.'s is used for estimating e.s.d.'s involving l.s. planes.
Refinement. Refinement of *F*^2^ against ALL reflections. The weighted *R*-factor *wR* and goodness of fit *S* are based on *F*^2^, conventional *R*-factors *R* are based on *F*, with *F* set to zero for negative *F*^2^. The threshold expression of *F*^2^ > σ(*F*^2^) is used only for calculating *R*-factors(gt) *etc*. and is not relevant to the choice of reflections for refinement. *R*-factors based on *F*^2^ are statistically about twice as large as those based on *F*, and *R*- factors based on ALL data will be even larger.

### Fractional atomic coordinates and isotropic or equivalent isotropic displacement parameters (Å^2^)

**Table:**

	*x*	*y*	*z*	*U*_iso_*/*U*_eq_	
S1	0.02806 (4)	0.24483 (3)	0.15884 (3)	0.02334 (9)	
O1	0.13544 (9)	0.55093 (8)	0.09394 (7)	0.0220 (2)	
O2	0.60961 (13)	−0.14734 (11)	0.22384 (10)	0.0532 (3)	
O3	0.68162 (11)	−0.25204 (9)	0.38191 (9)	0.0387 (3)	
N1	−0.10804 (11)	0.39532 (8)	0.28523 (8)	0.0151 (2)	
N2	−0.13256 (11)	0.41445 (9)	0.39629 (8)	0.0179 (2)	
N3	0.00696 (11)	0.25532 (8)	0.38914 (9)	0.0164 (2)	
N4	0.08649 (11)	0.15773 (9)	0.44203 (9)	0.0196 (2)	
N5	0.59545 (13)	−0.18126 (11)	0.33417 (11)	0.0313 (3)	
C1	−0.02269 (13)	0.29884 (10)	0.27574 (10)	0.0167 (2)	
C2	−0.06308 (13)	0.32779 (11)	0.45797 (11)	0.0181 (2)	
C3	−0.17203 (13)	0.47954 (10)	0.19116 (10)	0.0157 (2)	
H3	−0.1289	0.4604	0.1133	0.019*	
C4	−0.12191 (13)	0.60715 (10)	0.16885 (10)	0.0176 (3)	
H4A	−0.1706	0.6630	0.1094	0.021*	
H4B	−0.1595	0.6258	0.2465	0.021*	
C5	0.05068 (13)	0.62844 (11)	0.12070 (10)	0.0177 (3)	
C6	0.11439 (14)	0.74641 (11)	0.10917 (10)	0.0204 (3)	
C7	0.27293 (16)	0.75992 (13)	0.08526 (12)	0.0291 (3)	
H7	0.3383	0.6955	0.0748	0.035*	
C8	0.33498 (18)	0.86764 (14)	0.07670 (13)	0.0394 (4)	
H8	0.4428	0.8769	0.0607	0.047*	
C9	0.23910 (19)	0.96179 (13)	0.09158 (13)	0.0386 (4)	
H9	0.2818	1.0358	0.0845	0.046*	
C10	0.08236 (18)	0.94858 (12)	0.11653 (12)	0.0326 (3)	
H10	0.0174	1.0130	0.1278	0.039*	
C11	0.01941 (16)	0.84130 (11)	0.12527 (11)	0.0251 (3)	
H11	−0.0886	0.8325	0.1423	0.030*	
C12	−0.34582 (13)	0.46533 (10)	0.22760 (10)	0.0164 (2)	
C13	−0.43887 (14)	0.49889 (11)	0.32564 (11)	0.0207 (3)	
H13	−0.3932	0.5289	0.3723	0.025*	
C14	−0.59773 (14)	0.48880 (11)	0.35547 (11)	0.0237 (3)	
H14	−0.6601	0.5114	0.4227	0.028*	
C15	−0.66556 (14)	0.44607 (12)	0.28775 (11)	0.0255 (3)	
H15	−0.7743	0.4401	0.3078	0.031*	
C16	−0.57461 (15)	0.41212 (12)	0.19071 (12)	0.0278 (3)	
H16	−0.6209	0.3825	0.1442	0.033*	
C17	−0.41507 (14)	0.42135 (11)	0.16104 (11)	0.0228 (3)	
H17	−0.3531	0.3973	0.0946	0.027*	
C18	0.17967 (14)	0.10503 (11)	0.37509 (11)	0.0234 (3)	
H18	0.1924	0.1295	0.2895	0.028*	
C19	0.26734 (14)	0.00548 (11)	0.43263 (11)	0.0208 (3)	
C20	0.38127 (14)	−0.04456 (12)	0.35914 (12)	0.0244 (3)	
H20	0.3982	−0.0181	0.2730	0.029*	
C21	0.46964 (14)	−0.13341 (11)	0.41314 (12)	0.0229 (3)	
C22	0.44816 (14)	−0.17667 (11)	0.53782 (12)	0.0236 (3)	
H22	0.5108	−0.2379	0.5724	0.028*	
C23	0.33234 (15)	−0.12806 (11)	0.61123 (12)	0.0249 (3)	
H23	0.3142	−0.1568	0.6975	0.030*	
C24	0.24284 (14)	−0.03785 (11)	0.55959 (11)	0.0228 (3)	
H24	0.1641	−0.0051	0.6108	0.027*	
C25	−0.05494 (15)	0.30789 (12)	0.58367 (11)	0.0268 (3)	
H25A	−0.1045	0.3738	0.6121	0.040*	
H25B	0.0529	0.3048	0.5838	0.040*	
H25C	−0.1078	0.2319	0.6383	0.040*	

### Atomic displacement parameters (Å^2^)

**Table:**

	*U*^11^	*U*^22^	*U*^33^	*U*^12^	*U*^13^	*U*^23^
S1	0.03061 (18)	0.02282 (18)	0.02119 (17)	0.01031 (13)	−0.01063 (14)	−0.01219 (14)
O1	0.0183 (4)	0.0238 (5)	0.0240 (5)	0.0038 (3)	−0.0054 (4)	−0.0089 (4)
O2	0.0497 (7)	0.0763 (9)	0.0373 (6)	0.0366 (6)	−0.0175 (5)	−0.0258 (6)
O3	0.0318 (6)	0.0383 (6)	0.0484 (6)	0.0215 (5)	−0.0167 (5)	−0.0164 (5)
N1	0.0162 (5)	0.0169 (5)	0.0133 (5)	0.0032 (4)	−0.0047 (4)	−0.0063 (4)
N2	0.0187 (5)	0.0214 (5)	0.0154 (5)	0.0043 (4)	−0.0063 (4)	−0.0078 (4)
N3	0.0170 (5)	0.0156 (5)	0.0175 (5)	0.0048 (4)	−0.0072 (4)	−0.0053 (4)
N4	0.0190 (5)	0.0164 (5)	0.0241 (5)	0.0052 (4)	−0.0110 (4)	−0.0040 (4)
N5	0.0257 (6)	0.0334 (7)	0.0388 (7)	0.0122 (5)	−0.0128 (5)	−0.0160 (6)
C1	0.0159 (6)	0.0157 (6)	0.0179 (6)	0.0013 (4)	−0.0055 (5)	−0.0043 (5)
C2	0.0160 (6)	0.0206 (6)	0.0186 (6)	0.0029 (5)	−0.0051 (5)	−0.0078 (5)
C3	0.0162 (6)	0.0178 (6)	0.0137 (6)	0.0040 (4)	−0.0059 (5)	−0.0049 (5)
C4	0.0182 (6)	0.0174 (6)	0.0163 (6)	0.0040 (5)	−0.0047 (5)	−0.0052 (5)
C5	0.0201 (6)	0.0204 (6)	0.0124 (6)	0.0014 (5)	−0.0062 (5)	−0.0035 (5)
C6	0.0259 (7)	0.0223 (6)	0.0132 (6)	−0.0024 (5)	−0.0068 (5)	−0.0043 (5)
C7	0.0267 (7)	0.0344 (8)	0.0279 (7)	−0.0040 (6)	−0.0062 (6)	−0.0125 (6)
C8	0.0338 (8)	0.0462 (10)	0.0393 (9)	−0.0160 (7)	−0.0080 (7)	−0.0136 (7)
C9	0.0554 (10)	0.0269 (8)	0.0357 (8)	−0.0141 (7)	−0.0163 (7)	−0.0070 (7)
C10	0.0503 (9)	0.0218 (7)	0.0277 (7)	−0.0012 (6)	−0.0167 (7)	−0.0052 (6)
C11	0.0330 (7)	0.0209 (7)	0.0220 (7)	0.0007 (5)	−0.0110 (6)	−0.0050 (5)
C12	0.0165 (6)	0.0144 (6)	0.0172 (6)	0.0031 (4)	−0.0065 (5)	−0.0025 (5)
C13	0.0198 (6)	0.0227 (6)	0.0212 (6)	0.0040 (5)	−0.0075 (5)	−0.0082 (5)
C14	0.0192 (6)	0.0260 (7)	0.0223 (7)	0.0061 (5)	−0.0034 (5)	−0.0063 (6)
C15	0.0155 (6)	0.0282 (7)	0.0287 (7)	0.0017 (5)	−0.0071 (5)	−0.0033 (6)
C16	0.0244 (7)	0.0333 (8)	0.0301 (7)	−0.0019 (6)	−0.0128 (6)	−0.0111 (6)
C17	0.0220 (6)	0.0259 (7)	0.0221 (6)	0.0015 (5)	−0.0068 (5)	−0.0096 (6)
C18	0.0233 (7)	0.0250 (7)	0.0223 (7)	0.0071 (5)	−0.0092 (5)	−0.0072 (6)
C19	0.0194 (6)	0.0179 (6)	0.0273 (7)	0.0037 (5)	−0.0103 (5)	−0.0076 (5)
C20	0.0231 (7)	0.0271 (7)	0.0259 (7)	0.0067 (5)	−0.0108 (5)	−0.0103 (6)
C21	0.0195 (6)	0.0205 (6)	0.0320 (7)	0.0049 (5)	−0.0091 (5)	−0.0122 (6)
C22	0.0201 (6)	0.0161 (6)	0.0346 (7)	0.0027 (5)	−0.0125 (5)	−0.0044 (6)
C23	0.0241 (7)	0.0216 (7)	0.0256 (7)	0.0006 (5)	−0.0086 (5)	−0.0019 (6)
C24	0.0205 (6)	0.0202 (6)	0.0273 (7)	0.0029 (5)	−0.0071 (5)	−0.0076 (6)
C25	0.0315 (7)	0.0312 (7)	0.0220 (7)	0.0127 (6)	−0.0133 (6)	−0.0117 (6)

### Geometric parameters (Å, °)

**Table:**

S1—C1	1.6656 (12)	C10—C11	1.3870 (18)
O1—C5	1.2176 (14)	C10—H10	0.9500
O2—N5	1.2278 (14)	C11—H11	0.9500
O3—N5	1.2244 (14)	C12—C17	1.3878 (17)
N1—C1	1.3488 (14)	C12—C13	1.3952 (16)
N1—N2	1.3828 (12)	C13—C14	1.3880 (17)
N1—C3	1.4765 (14)	C13—H13	0.9500
N2—C2	1.2975 (15)	C14—C15	1.3824 (18)
N3—C2	1.3802 (14)	C14—H14	0.9500
N3—N4	1.3881 (13)	C15—C16	1.3820 (17)
N3—C1	1.3944 (14)	C15—H15	0.9500
N4—C18	1.2694 (14)	C16—C17	1.3933 (17)
N5—C21	1.4697 (16)	C16—H16	0.9500
C2—C25	1.4801 (16)	C17—H17	0.9500
C3—C12	1.5187 (15)	C18—C19	1.4676 (16)
C3—C4	1.5202 (16)	C18—H18	0.9500
C3—H3	1.0000	C19—C20	1.3884 (17)
C4—C5	1.5152 (15)	C19—C24	1.4010 (17)
C4—H4A	0.9900	C20—C21	1.3815 (17)
C4—H4B	0.9900	C20—H20	0.9500
C5—C6	1.4955 (17)	C21—C22	1.3793 (17)
C6—C11	1.3944 (17)	C22—C23	1.3875 (17)
C6—C7	1.3963 (17)	C22—H22	0.9500
C7—C8	1.3890 (19)	C23—C24	1.3866 (17)
C7—H7	0.9500	C23—H23	0.9500
C8—C9	1.388 (2)	C24—H24	0.9500
C8—H8	0.9500	C25—H25A	0.9800
C9—C10	1.378 (2)	C25—H25B	0.9800
C9—H9	0.9500	C25—H25C	0.9800
			
C1—N1—N2	113.68 (9)	C10—C11—H11	119.9
C1—N1—C3	127.18 (10)	C6—C11—H11	119.9
N2—N1—C3	119.13 (9)	C17—C12—C13	118.78 (11)
C2—N2—N1	104.77 (9)	C17—C12—C3	120.07 (10)
C2—N3—N4	117.92 (9)	C13—C12—C3	121.13 (10)
C2—N3—C1	108.76 (9)	C14—C13—C12	120.45 (12)
N4—N3—C1	133.31 (9)	C14—C13—H13	119.8
C18—N4—N3	119.73 (10)	C12—C13—H13	119.8
O3—N5—O2	123.47 (11)	C15—C14—C13	120.29 (12)
O3—N5—C21	118.13 (11)	C15—C14—H14	119.9
O2—N5—C21	118.39 (11)	C13—C14—H14	119.9
N1—C1—N3	102.22 (9)	C16—C15—C14	119.79 (12)
N1—C1—S1	128.11 (9)	C16—C15—H15	120.1
N3—C1—S1	129.64 (9)	C14—C15—H15	120.1
N2—C2—N3	110.56 (10)	C15—C16—C17	120.06 (12)
N2—C2—C25	126.08 (11)	C15—C16—H16	120.0
N3—C2—C25	123.34 (10)	C17—C16—H16	120.0
N1—C3—C12	110.99 (9)	C12—C17—C16	120.62 (12)
N1—C3—C4	109.66 (9)	C12—C17—H17	119.7
C12—C3—C4	111.73 (9)	C16—C17—H17	119.7
N1—C3—H3	108.1	N4—C18—C19	118.61 (11)
C12—C3—H3	108.1	N4—C18—H18	120.7
C4—C3—H3	108.1	C19—C18—H18	120.7
C5—C4—C3	113.55 (10)	C20—C19—C24	118.98 (11)
C5—C4—H4A	108.9	C20—C19—C18	119.20 (11)
C3—C4—H4A	108.9	C24—C19—C18	121.78 (11)
C5—C4—H4B	108.9	C21—C20—C19	119.02 (12)
C3—C4—H4B	108.9	C21—C20—H20	120.5
H4A—C4—H4B	107.7	C19—C20—H20	120.5
O1—C5—C6	120.86 (11)	C22—C21—C20	122.89 (11)
O1—C5—C4	120.93 (11)	C22—C21—N5	118.42 (11)
C6—C5—C4	118.20 (10)	C20—C21—N5	118.66 (11)
C11—C6—C7	119.51 (12)	C21—C22—C23	117.96 (11)
C11—C6—C5	121.74 (11)	C21—C22—H22	121.0
C7—C6—C5	118.72 (11)	C23—C22—H22	121.0
C8—C7—C6	119.97 (14)	C24—C23—C22	120.48 (12)
C8—C7—H7	120.0	C24—C23—H23	119.8
C6—C7—H7	120.0	C22—C23—H23	119.8
C9—C8—C7	119.85 (14)	C23—C24—C19	120.66 (11)
C9—C8—H8	120.1	C23—C24—H24	119.7
C7—C8—H8	120.1	C19—C24—H24	119.7
C10—C9—C8	120.45 (13)	C2—C25—H25A	109.5
C10—C9—H9	119.8	C2—C25—H25B	109.5
C8—C9—H9	119.8	H25A—C25—H25B	109.5
C9—C10—C11	120.07 (14)	C2—C25—H25C	109.5
C9—C10—H10	120.0	H25A—C25—H25C	109.5
C11—C10—H10	120.0	H25B—C25—H25C	109.5
C10—C11—C6	120.13 (13)		
			
C1—N1—N2—C2	−0.75 (13)	C8—C9—C10—C11	−0.9 (2)
C3—N1—N2—C2	−179.67 (10)	C9—C10—C11—C6	0.17 (19)
C2—N3—N4—C18	−164.43 (11)	C7—C6—C11—C10	0.54 (18)
C1—N3—N4—C18	16.93 (19)	C5—C6—C11—C10	178.58 (11)
N2—N1—C1—N3	0.20 (12)	N1—C3—C12—C17	−113.03 (12)
C3—N1—C1—N3	179.01 (10)	C4—C3—C12—C17	124.22 (12)
N2—N1—C1—S1	178.48 (9)	N1—C3—C12—C13	68.72 (13)
C3—N1—C1—S1	−2.70 (18)	C4—C3—C12—C13	−54.02 (14)
C2—N3—C1—N1	0.41 (12)	C17—C12—C13—C14	−0.26 (17)
N4—N3—C1—N1	179.13 (11)	C3—C12—C13—C14	178.01 (10)
C2—N3—C1—S1	−177.84 (10)	C12—C13—C14—C15	−0.43 (18)
N4—N3—C1—S1	0.88 (19)	C13—C14—C15—C16	0.66 (19)
N1—N2—C2—N3	0.99 (13)	C14—C15—C16—C17	−0.21 (19)
N1—N2—C2—C25	179.86 (11)	C13—C12—C17—C16	0.71 (18)
N4—N3—C2—N2	−179.87 (10)	C3—C12—C17—C16	−177.57 (11)
C1—N3—C2—N2	−0.92 (13)	C15—C16—C17—C12	−0.48 (19)
N4—N3—C2—C25	1.22 (17)	N3—N4—C18—C19	177.32 (10)
C1—N3—C2—C25	−179.83 (11)	N4—C18—C19—C20	−173.19 (12)
C1—N1—C3—C12	110.32 (12)	N4—C18—C19—C24	4.59 (18)
N2—N1—C3—C12	−70.93 (12)	C24—C19—C20—C21	−1.64 (19)
C1—N1—C3—C4	−125.75 (12)	C18—C19—C20—C21	176.20 (12)
N2—N1—C3—C4	53.00 (13)	C19—C20—C21—C22	1.2 (2)
N1—C3—C4—C5	61.96 (12)	C19—C20—C21—N5	−176.71 (11)
C12—C3—C4—C5	−174.54 (9)	O3—N5—C21—C22	−4.44 (18)
C3—C4—C5—O1	5.85 (15)	O2—N5—C21—C22	176.29 (13)
C3—C4—C5—C6	−173.01 (9)	O3—N5—C21—C20	173.56 (12)
O1—C5—C6—C11	172.26 (11)	O2—N5—C21—C20	−5.71 (19)
C4—C5—C6—C11	−8.89 (16)	C20—C21—C22—C23	0.00 (19)
O1—C5—C6—C7	−9.69 (17)	N5—C21—C22—C23	177.90 (11)
C4—C5—C6—C7	169.17 (10)	C21—C22—C23—C24	−0.70 (19)
C11—C6—C7—C8	−0.52 (19)	C22—C23—C24—C19	0.22 (19)
C5—C6—C7—C8	−178.62 (11)	C20—C19—C24—C23	0.97 (18)
C6—C7—C8—C9	−0.2 (2)	C18—C19—C24—C23	−176.82 (12)
C7—C8—C9—C10	0.9 (2)		

### Hydrogen-bond geometry (Å, °)

**Table:**

*D*—H···*A*	*D*—H	H···*A*	*D*···*A*	*D*—H···*A*
C3—H3···O1^i^	1.00	2.57	3.4922 (15)	154
C4—H4B···O3^ii^	0.99	2.59	3.5002 (16)	153
C17—H17···O1^i^	0.95	2.47	3.3076 (15)	147
C25—H25B···O3^iii^	0.98	2.57	3.5310 (18)	168

Symmetry codes: (i) −*x*, −*y*+1, −*z*; (ii) *x*−1, *y*+1, *z*; (iii) −*x*+1, −*y*, −*z*+1.
